# Bearing Fault Diagnosis Considering the Effect of Imbalance Training Sample

**DOI:** 10.3390/e21040386

**Published:** 2019-04-10

**Authors:** Lin Lin, Bin Wang, Jiajin Qi, Da Wang, Nantian Huang

**Affiliations:** 1 College of Information and Control Engineering, Jilin Institute of Chemical Technology, Jilin 132022, China; 2 Taian Power Supply Company, State Grid Shandong Electric Power Co. Ltd., Taian 271000, China; 3 Hangzhou Municipal Electric Power Supply Company of State Grid, Hangzhou 310009, China; 4 Dezhou Power Supply Company, State Grid Shandong Electric Power Co. Ltd., Dezhou 253000, China; 5 School of Electrical Engineering, Northeast Electric Power University, Jilin 132012, China

**Keywords:** bearing fault diagnosis, empirical wavelet transform, one-class support vector machine, random forest, imbalanced training data

## Abstract

To improve the accuracy of the recognition of complicated mechanical faults in bearings, a large number of features containing fault information need to be extracted. In most studies regarding bearing fault diagnosis, the influence of the limitation of fault training samples has not been considered. Furthermore, commonly used multi-classifiers could misidentify the type or severity of faults without using normal samples as training samples. Therefore, a novel bearing fault diagnosis method based on the one-class classification concept and random forest is proposed for reducing the impact of the limitations of the fault training sample. First, the bearing vibration signals are decomposed into numerous intrinsic mode functions using empirical wavelet transform. Then, 284 features including multiple entropy are extracted from the original signal and intrinsic mode functions to construct the initial feature set. Lastly, a hybrid classifier based on one-class support vector machine trained by normal samples and a random forest trained by imbalanced fault data without some specific severities is set up to accurately identify the mechanical state and specific fault type of the bearings. The experimental results show that the proposed method can significantly improve the classification accuracy compared with traditional methods in different diagnostic target.

## 1. Introduction

Bearings are one of the most important components in rotating machinery, and a bearing fault can affect the reliability of wind turbines or other electric equipment. A gearbox fault or other mechanical fault in the drive system of wind turbines is mostly caused by a bearing fault or is reflected in the state of the bearings. Therefore, research into bearing fault diagnosis is crucial for improving the electronic reliability of electrical equipment and reducing downtime [1,2,3].

Vibration analysis has been widely used in the field of bearing fault diagnosis [4,5,6,7]. However, it is difficult to extract features from raw vibration signals with nonlinear and non-stationary characteristics, and thus, the raw signal needs to be pre-processed using time-frequency analysis methods. The commonly used methods for this pre-processing include empirical mode decomposition (EMD) [7,8], wavelet packet transform (WPT) [9], local mean decomposition (LMD) [6], and ensemble empirical mode decomposition (EEMD) [10]. These methods are effective, but have a few limitations. WPT is not self-adaptive, and the various selections of the wavelet basis function would seriously affect the obtained results. EMD and LMD have limitations such as end effect and mode confusion [11]. In the case of EEMD, mode confusion is overcome by adding white noise to the original signal; however, the computation is also greatly increased. Compared to other methods, empirical wavelet transform (EWT) [12] has been proven to provide more stable decomposition results with less computation than the aforementioned methods. EWT has thus been applied preliminarily in fault diagnosis and has achieved good results [13,14]. 

After processing the raw bearing signals, the primary objective is to extract efficient features. The work of Rai et al. in [15] extracts the singular value and energy-entropy features from intrinsic mode functions (IMFs) obtained using EMD for bearing performance degradation assessment. Bustos et al. [8] proves the validity of EMD and average PSD method for the identification of the bogie operating state of high-speed train. It is also applicable to any other mechanical system as well. Only singular value features extracted from the components decomposed using LMD are used to achieve bearing fault diagnosis in [6]. Multi-scale entropy is extracted from IMFs obtained using EEMD for bearing diagnosis in [10]. Statistical features are extracted for fault classification in [16,17]. Comprehensive features should be extracted to avoid missing important information. However, extracting a large number of features would cause a great increase in feature dimensions, which will decrease the performance of the classification systems in terms of accuracy [17,18,19]. Therefore, feature selection is crucial to improving the performance of the system [20].

Essentially, feature selection can be divided into wrappers, filters, and embedded methods [21,22,23]. Wrappers use the predictive power of a learning machine to assess the feature subsets; these types of methods have a small deviation, but the massive amounts of computation required makes wrappers unsuitable for processing large data. Usually, as compared to wrappers, filters are faster, but may provide poor performance. For embedded learning methods such as classification and regression tree (CART) and random forest (RF) [24,25,26], feature selection is incorporated in the training process of the model. Therefore, embedded methods would be more efficient than wrappers and more precise than filters, and RF has better robustness than CART [20]. Mahapatra discards the feature elements with minimal influence on the classification performance by RF and achieves better generalization of the RF-based classifier [26]. Although feature selection can improve classification efficiency and the accuracy of the classifier, it needs to be carried out under specific classification targets, and the feature selection results are different with different training data sets and classification targets. Without considering the particularity of related applications, the classifier constructed after feature selection is prone to over-fitting.

The feature set is input into the classifier to achieve automatic fault diagnosis. No classifier is used in [13,27,28]; the fault identification is performed by using peak visualization in frequency graphs, which is called envelope analysis. Castejon realized effective identification of four kinds of bearing faults by an automatic fault classification technique based on multi-resolution analysis and neural networks in a real industry [3]. Envelope analysis requires prior knowledge regarding the fault characteristic frequency of bearings. Therefore, using machine-learning models such as support vector machines (SVMs), back propagation neural networks (BPNNs), and extreme learning machines (ELMs) for achieving automatic fault diagnosis is the primary method used in contemporary work [1].

Moreover, deep learning algorithms such as deep neural networks (DNNs) [2], deep belief networks (DBNs) [7] and convolutional neural networks (CNNs) [9] have attracted wide attention in the gearbox and bearing fault diagnosis recently. Because of the outstanding ability to extract high-level features from raw data, the models based on deep learning can obtain superior accuracy. However, it is still difficult to optimize the complex structure and characters of deep learning methods.

Methods based on traditional machine-learning or deep learning have been successfully applied in fault diagnosis [1,2,3,4,5,6,7,8,9]. They only use normal samples and historical fault samples to train multi-classifiers for fault diagnosis. However, the applications of all of these methods have not considered the limitations of the imbalance of training samples. In this paper, the imbalance of sample number is due to the fact that a certain number of fault samples have been obtained after the occurrence of certain types of bearing fault, while some fault severities, which have not happened or cannot be obtained by experiment for reasons of cost, have no accumulation of samples.

Moreover, when a fault degree that is not included in the training dataset occurs, the diagnosis system may mistake it for a normal condition. Therefore, utilizing only normal samples as training samples for precisely distinguishing the fault condition of bearings has good practical value [22]. Wan designed a hybrid classifier with good diagnostic results for preventing the misidentification of unknown faults [11].

Otherwise, the diagnosis methods should fully consider the effect of diagnostic targets. Most of the studies develop bearing-fault diagnosis for three common faults that are classified by the fault location: ball fault, inner race fault, and outer race fault [2,22]. However, the requirements for various scenarios are not the same in application. Thus, further refinement of bearing fault types is required. In addition to the fault location, the position of the bearing and the fault severity can both be regarded as specific fault types [1]. For different diagnostic targets, the optimal feature subset is different. This leads to difficulty in constructing classifiers with optimal feature subsets.

A novel bearing fault diagnosis method based on a hybrid classifier constructed using one-class classification and RF considering the imbalance of the training sample is proposed. EWT is used to extract the IMFs of the bearing vibration signal; 284 features are extracted from the original signal and IMFs to construct the initial feature set. One-class support vector machine (OCSVM), trained using only normal samples in the hybrid classifier, is used to determine if a bearing fault has occurred. The classifier based on RF with the original feature set is applied for fault diagnosis with unbalanced training samples, and the influence of redundant features is avoided in the ensemble learning process of RF. If a severe fault does occur, the RF trained with all known fault severities is used to recognize the specific fault type. The experimental results show that the new method can improve the identification accuracy of the mechanical fault type severity samples not included in the training samples and provides a superior result for bearing fault diagnosis.

## 2. Empirical Wavelet Transform

EWT overcomes the shortcomings of theory and mode mixing in EMD [12]. In this method, an orthogonal wavelet filter bank is constructed, by which amplitude modulated-frequency modulated (AM-FM) components with a compactly supported Fourier spectrum are extracted. These AM-FM components can describe the intrinsic modes of the original vibration signal. So, like EMD, EWT can decompose the original bearing vibration signal f(t) into a series of IMFs denoted by $f_{k}(t)$. Therefore,

(1) $$f(t)=\sum_{k=0}^{L}f_{k}(t)$$

where each $f_{k}(t)$ is an AM-FM function. 

The process of EWT includes the following three steps:

Step 1: Process the original bearing signal via Fast Fourier transform (FFT).

Step 2: Adaptively segment the Fourier spectrum of the signal.

Step 3: Apply scaling and wavelet functions corresponding to each segment to generate bandpass filters on each segment.

In [12], Gilles referred to the construction of both Littlewood-Paley and Meyer’s wavelets. To choose the appropriate wavelet filter banks, the Fourier spectrum must be split adaptively. Suppose the Fourier support [0,π] is split into N successive parts. Then, $\omega_{l}(l=1,2,\cdots ,N)$ represents the boundaries of the parts. An empirical scaling function ${\hat{\phi }}_{l}(\omega )$ and the empirical wavelets ${\hat{\psi }}_{l}(\omega )$ are defined by Expressions (2) and (3), respectively. 

(2) $${\hat{\phi }}_{l}\left(\omega \right)=\{\begin{array}{l} 1,\;\mathrm{if}\;\left|\omega \right|\leq (1-\gamma )\omega_{l}\\ \begin{array}{l} \cos\left[\frac{\pi }{2}\beta \left(\frac{1}{2\gamma \omega_{l}}\left(\left|\omega \right|-\left(1-\gamma \right)\omega_{l}\right)\right)\right],\\ \;\mathrm{if}\;(1-\gamma )\;\omega_{l}\leq \left|\omega \right|\leq (1+\gamma )\omega_{l} \end{array}\\ 0,\;\mathrm{otherwise} \end{array}$$

(3) $${\hat{\psi }}_{l}\left(\omega \right)=\{\begin{array}{l} \begin{array}{l} \begin{array}{l} 1,\;\mathrm{if}(1+\gamma )\omega_{l}\leq \left|\omega \right|\leq (1-\gamma )\omega_{l+1}\\ \cos\left[\frac{\pi }{2}\beta \left(\frac{1}{2\gamma \omega_{l+1}}\left(\left|\omega \right|-(1-\gamma )\omega_{l+1}\right)\right)\right], \end{array}\\ \;\mathrm{if}\;(1-\gamma )\omega_{l+1}\leq \left|\omega \right|\leq (1+\gamma )\omega_{l+1} \end{array}\\ \sin\left[\frac{\pi }{2}\beta \left(\frac{1}{2\gamma \omega_{l}}\left(\left|\omega \right|-(1-\gamma )\omega_{l}\right)\right)\right],\;\\ \;\mathrm{if}\;(1-\gamma )\omega_{l}\leq \left|\omega \right|\leq (1+\gamma )\omega_{l}\\ 0,\;\mathrm{otherwise}\; \end{array}$$

EWT is defined like the classic wavelet transform. If F[⋅] and $F^{-1}[\cdot ]$ represent the Fourier transform and its inverse transform, the detail coefficients are obtained by the inner products of applied signal with the empirical wavelets:

(4) $$W_{f}^{e}(l,t)=\langle f,\psi_{l}\rangle =\int f(\tau )\bar{\psi_{l}(\tau -t)}d\tau =F^{-1}[f(\omega ){\hat{\psi }}_{l}(\omega )]\;$$

The approximation coefficients are obtained by the inner product of the applied signal with the scaling function:

(5) $$W_{f}^{e}(0,t)=\langle f,\phi_{1}\rangle =\int f(\tau )\bar{\phi_{1}(\tau -t)}d\tau =F^{-1}[f(\omega ){\hat{\phi }}_{1}(\omega )]\;$$

where ${\hat{\psi }}_{l}(\omega )$ and ${\hat{\phi }}_{l}(\omega )$ represent the Fourier transform of $\psi_{l}(\omega )$ and ${\hat{\phi }}_{l}(\omega )$, respectively. $\bar{\psi_{l}(t)}$ and $\bar{\phi_{l}(t)}$ represent the complex conjugate of $\psi_{l}(t)$ and $\phi_{l}(t)$, respectively. Then, the empirical mode $f_{k}(t)$ of the bearing vibration signal in (1) can be obtained by

(6) $$f_{0}(t)=W_{f}^{e}(0,t)*\phi_{l}(t)$$

(7) $$f_{k}(t)=W_{f}^{e}(k,t)*\psi_{k}(t)$$

## 3. Construction and Classification Progress of RF

RF is a classification algorithm based on a collection of decision trees built using a bootstrap sample. For tree building, both bagging and random feature selection are used in this method. Compared with SVM and ELM, RF has a superior classification ability [25]. The main characteristics of RF are strong robustness to outliers and noise, effective in assessing the generalization error, strength, correlation, and feature importance, and effective in preventing over-fitting [26]. The detailed classification principle of RF can be found in [26], and its classification process is as follows:

(1) *K* sample sets selected randomly with replacement by bootstrap are used to build *K* decision trees, and the remaining samples after every selection are regarded as out-of-bag data.
(2) $m_{try}$ features are selected from each node of the decision trees. The amount of discriminative information contained in the features is used to estimate the classification ability of the different features. The feature with the strongest classification ability is regarded as a segmentation feature of the node. Usually, $m_{try}=\sqrt{M}$, where *M* is the total number of features.
(3) To obtain low-bias trees, no pruning operation is performed in each tree.
(4) RF is constructed with *K* decision trees obtained through the above process. For the tested bearing samples, the final classification result of RF is determined by taking the voting results of all decision trees into account.

## 4. One-Class Support Vector

Because of the advantage of being trained by only one type of target sample, one-class classification can make up for the shortcoming of excessive reliance on the training samples in the multi-class classifiers. OCSVM is suitable for solving small sample, high dimension and non-linear problems. In this paper, OCSVM is used in the monitoring of bearing conditions.

For a given training set $\left\{x_{i}\right\},\;i=1,2,\ldots ,N$, N represents the sample number in the training set. The aim of OCSVM is to find a hyperplane f(x)=〈ω,x〉−ρ that can separate the target samples (that is, the normal samples of bearings) and an origin with a maximal margin in a high-dimensional feature space [29]. The parameters ω and ρ are used to express the normal vector and intercept of the hyperplane, respectively. A slack variable $\xi_{i}$ is introduced to allow some outliers in training samples. v∈(0,1] is called the error limitation, which is used to control the upper limit on the number of outliers. Nonlinear mapping ψ:x→ψ(x) can map the samples in input space to a high-dimensional feature space, coming down to the following quadratic programming problem:

(8) $$\left\{ \begin{array}{l} \min\frac{1}{2}\omega^{2}+\frac{1}{vN}\overset{N}{\underset{i=1}{\sum }}\xi_{i}-\rho \\ s.t.\;\left(\omega ,\psi (x)\right)\geq \rho -\xi_{i}\;,\xi_{i}\geq 0 \end{array}\right.\;$$

By introducing the kernel function and Lagrange multiplier $\alpha_{i}$, Equation (8) is transformed into

(9) $$\left\{ \begin{array}{l} \min\frac{1}{2}\overset{N}{\underset{i=1}{\sum }}\overset{N}{\underset{j=1}{\sum }}\alpha_{i}\alpha_{j}K(x_{i},x_{j})\\ s.t.\;0\leq \alpha_{i}\leq \frac{1}{vN}\;,\;\overset{N}{\underset{i=1}{\sum }}\alpha_{i}=1 \end{array}\right.$$

Here, kernel function $K(x_{i},x_{j})=\langle \psi (x_{i}),\psi (x_{j})\rangle$. The Radial Basis Function (RBF) kernel function used in this paper is as follows, and σ represents the width of the kernel function.

(10) $$K\left(x_{i},x_{j}\right)=\exp\left\{-\frac{{\left\|x_{i}-x_{j}\right\|}^{2}}{2\sigma^{2}}\right\}$$

The decision function used to judge the state of bearings can be determined after obtaining $\alpha_{i}$ according to (11).

(11) $$f(z)=\mathrm{sgn}\left(\overset{N}{\underset{i=1}{\sum }}\alpha_{i}K(x_{i},z)-\overset{N}{\underset{i=1}{\sum }}\alpha_{i}K(x_{i},x_{j})\right)$$

After training OCSVM, for any bearing vibration sample *z*, whether *z* is a fault sample can be determined by (11).

**Figure 1:**
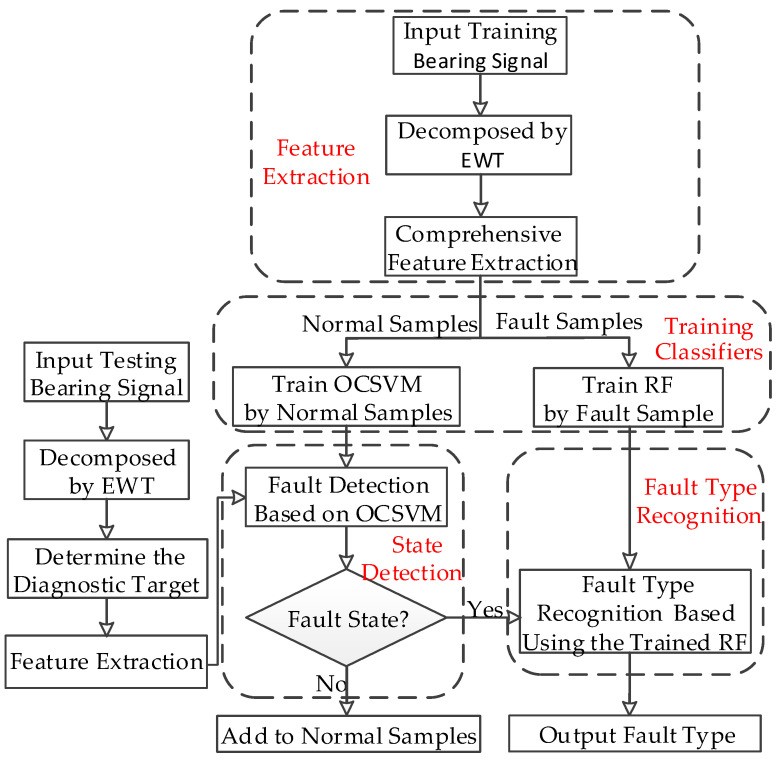
The flowchart of the proposed method.

The proposed method includes feature extraction, the training of the classifier, state detection and fault type recognition. First, EWT is used to extract the IMFs of the bearing vibration signal. Then, 284 features are extracted from the original signal and IMFs to construct the initial feature set. Lastly, a hybrid classifier based on OCSVM and RF is set up. OCSVM is used to determine whether a bearing fault has occurred. If a fault has occurred, the RF trained with all known faults is used to recognize the specific fault type. The flowchart of the proposed method is shown in Figure 1.

## 5. Construction of the Initial Feature Set

**Figure 2:**
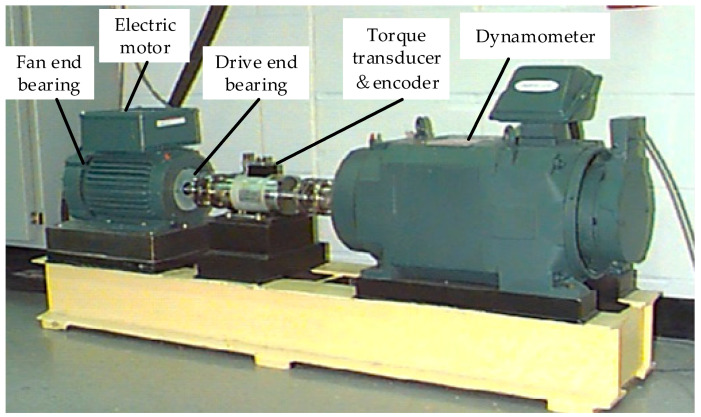
CWRU bearing test rig.

The bearing dataset provided by Case Western Reserve University (CWRU) [1] has been used as benchmark data in the field of bearing fault diagnosis. Thus, this dataset is chosen as the test data for verifying the proposed method. The basic layout of the test rig is shown in Figure 2. It consists of a 2 hp Reliance Electric motor driving a shaft on which a torque transducer and encoder are mounted. Torque is applied to the shaft via a dynamometer and electronic control system. For the tests, faults were seeded on the drive- and fan-end bearings (SKF deep-groove ball bearings: 6205-2RS JEM and 6203-2RS JEM, respectively) of the motor using electro-discharge machining (EDM). The faults were seeded on the rolling elements and on the inner and outer races, and each faulty bearing was reinstalled (separately) on the test rig, which was then run at constant speed for motor loads of 0–3 horsepower (approximate motor speeds of 1797–1730 rpm) [30]. The sampling frequency of fault data used in the paper was 12,000 points per second for bearing fault diagnosis in the experiment.

### 5.1. Condition Classes of the Experimental Data

The bearing dataset of CWRU provides the machine condition information containing different bearing fault locations (ball, inner race and outer race), the fault severity (i.e., 0.007, 0.014 and 0.021 mils in the diameter of the artificially drilled hole into the bearing) and the position of the motor bearing (drive end and fan end). Therefore, the database divided based on different diagnostic targets is used to demonstrate the validity of the method. When only identifying the bearing fault locations, the machine condition contains the normal condition and three types of faults: ball fault, inner race fault, and outer race fault. When identifying both the fault locations and the position of the bearing, the machine condition contains the normal condition and six types of faults, which include the ball fault at the drive and fan ends, inner race fault at the drive and fan ends, and outer race fault at the drive and fan ends. When considering the bearing fault locations, the position of the bearing and the three types of fault severity simultaneously, the machine condition can further be divided into a normal condition and eighteen types of faults [1].

The total duration of the signals in the CWRU database is approximately 10 s. To acquire more samples, the total duration can be divided into a series of successive intervals that can be regarded as independent patterns. For various studies on bearing fault diagnosis, the length of each interval varies from 1024 to 8000 points [1,6]. In general, the more sampling points in a signal, the more fault information is contained, which is more useful for improving the classification accuracy. However, considering the efficiency of feature extraction, the number of samples required and the number of sampling points in the relevant literature, the length of each sample is confirmed as 4096 points (that is, almost ten rotation periods) [1]. Finally, considering the locations of the bearing faults, the fault severity, and the position of the bearing, 2000 samples comprising 200 normal samples are obtained.

### 5.2. Analysis of the Bearing Vibration Signal Using EWT

**Figure 3:**
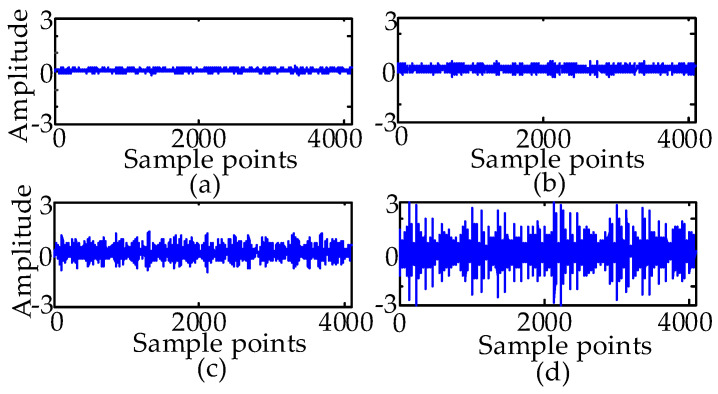
(**a**) Time domain waveform of the normal signal. (**b**) Time domain waveform of the ball fault signal at 0.007 mils. (**c**) Time domain waveform of the inner race fault signal at 0.007 mils. (**d**) Time domain waveform of the outer race fault signal at 0.007 mils.

Figure 3 lists the normal, ball fault, inner race fault, and outer race fault signal waveform with 0 hp acquired at the drive end (DE) with a diameter of 0.007 mils. The effective identifying information is submerged in noise. Therefore, EWT is used to extract effective features.

**Figure 4:**
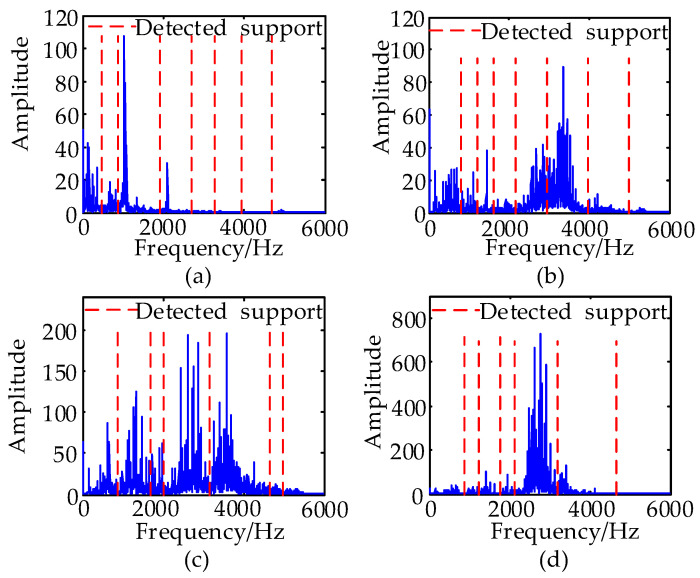
(**a**) The segmentation of the frequency spectrum of the normal signal. (**b**) The segmentation of the frequency spectrum of the ball fault signal at 0.007 mils. (**c**) The segmentation of the frequency spectrum of the inner race fault signal at 0.007 mils. (**d**) The segmentation of the frequency spectrum of the outer race fault signal at 0.007 mils.

**Figure 5:**
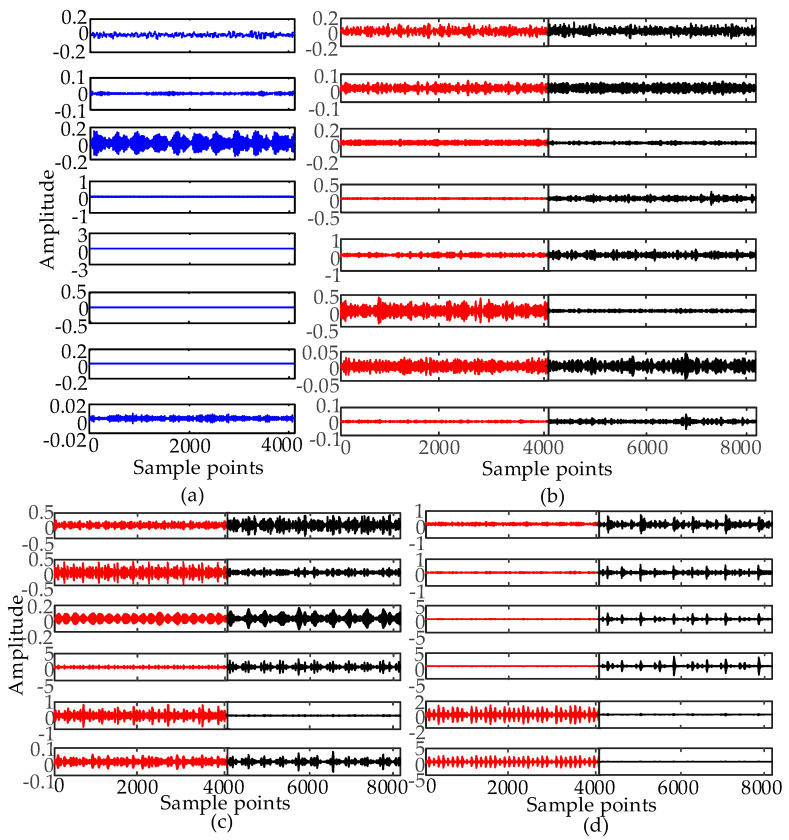
The EWT results of four types of signals at 0.007 mils and 0.021 mils. The fault severity of the signals in red is 0.007 mils. The fault severity of the signals in black is 0.021 mils. (**a**) EWT results of the normal signal. (**b**) EWT results of the ball fault signal. (**c**) EWT results of the inner race fault signal. (**d**) EWT results of the outer race fault signal.

The segmentation of the frequency spectrum of four types of bearing signals at 0.007 mils and decomposition results obtained by EWT are shown in Figure 4 and Figure 5, respectively. To observe the change in amplitude for each fault type with different severities, IMFs at normal, 0.007 and 0.021 mils are included in Figure 5.

In Figure 4, the Fourier spectrum of the original signal is divided into various regions, which denote the frequency range of the corresponding IMF in Figure 5. The components at 0 to 4000 Hz comprise a great percentage of the total signal in a ball fault and inner race fault. The main part of the normal signal is concentrated below 2000 Hz, showing that the energy distribution in different frequency bands of different types of fault vibration signals is different. From Figure 5, the amplitude of every IMF of the four types of signals has a greater difference at the same severity. The amplitudes of most of the IMFs for each fault type at different fault severities also have a greater difference. For the ball fault at 0.007 mils, the maximum amplitude appears in the sixth IMF, which is close to 0.5. For the ball fault at 0.021 mils, the maximum amplitude appears at the fifth IMF, which is close to 0.4. For the inner race fault at 0.007 mils, the maximum amplitude appears in the fifth IMF, which is close to 1. For the inner race fault at 0.021 mils, the maximum amplitude appears in the fourth IMF, which is close to 2. For the outer race fault at 0.007 mils, the maximum amplitude appears in the sixth IMF, which is close to 3. For the outer race fault at 0.021 mils, the maximum amplitude appears in the fourth IMF, which is close to 3. 

When the bearing failure emerges, the fault characters in the frequency distribution of the fault vibration signals will have changed, and the energy distribution in different frequency bands will show the corresponding change. EWT can decompose a multicomponent signal into some IMFs in different frequency bands. The experimental results shown in Figure 4 and Figure 5 prove that, by observing the amplitude of the IMFs and computing the energy distribution in different frequency bands and time domains, the features of different fault types of bearings can be extracted from EWT results. To accurately describe the fault characteristics and identify the fault type such as fault severity, more fault information should be mined from the raw signals and IMFs, and the integral fault diagnosis system can be constructed on those features.

However, owing to the complexity and the nature of various types of signals, the number of IMFs obtained from various fault signals using EWT may be different. After the statistical analysis, the usual number of IMFs is six to nine for various signals, and different IMFs contain different characters of time-frequency energy distribution for fault diagnosis. On observing the Fourier spectrum of the various signals in Figure 4 and the EWT results in Figure 5, we can observe that the energy distribution of four types of signals is concentrated mainly in the low-frequency and medium-frequency portions. Therefore, the normalized energy, which is the ratio of the energy of each IMF component to the energy of the raw signal, is treated as the selection criterion for useful IMF. To increase the persuasive power, the average energy ratio of 600 signals per type is calculated. Statistical analysis shows that the five IMF components with the most energy contain over 96% of the discriminative information. Therefore, the five IMF components with the most energy are selected as effective components for feature extraction. 

If the extracted features have a high sensitivity in the case of mechanical state changes of bearings under various operating conditions, the fault diagnosis capability of the systems can be enhanced greatly. The descriptive ability of features in the time and frequency domain has their own significance, and thus, synthetic analysis is required. Therefore, the numerous time and frequency domain features are extracted both from raw signals and IMFs to avoid missing important information. 

(1) Time domain: Eighteen types of time-domain features including maximum amplitude value (F*_y_*_,1_ and F*_y_*_,19_), minimum amplitude value (F*_y_*_,2_ and F*_y_*_,20_), mean value (F*_y_*_,3_ and F*_y_*_,21_), standard deviation (F*_y_*_,4_ and F*_y_*_,22_), absolute average (F*_y_*_,5_ and F*_y_*_,23_), skewness value(F*_y_*_,6_ and F*_y_*_,24_), kurtosis value (F*_y_*_,7_ and F*_y_*_,25_), peak-to-peak value (F*_y_*_,8_ and F*_y_*_,26_), square root of the amplitude (F*_y_*_,9_ and F*_y_*_,27_), root mean square (F*_y_*_,10_ and F*_y_*_,28_), peak value (F*_y_*_,11_ and F*_y_*_,29_), shape factor (F*_y_*_,12_ and F*_y_*_,30_), crest factor (F*_y_*_,13_ and F*_y_*_,31_), impulse factor (F*_y_*_,14_ and F*_y_*_,32_), margin factor (F*_y_*_,15_ and F*_y_*_,33_), skewness factor (F*_y_*_,16_ and F*_y_*_,34_), coefficient of variation (F*_y_*_,17_ and F*_y_*_,35_) and kurtosis factor (F*_y_*_,18_ and F*_y_*_,36_) are used. Here, y=0,1,2,⋯,5. When *y* = 0, the features are extracted from the raw signal; otherwise, the features are extracted from the *y*^th^ IMF. The same is shown below.

For the CWRU bearing data, the sensor installed at the fan end (FE) can detect the bearing faults at the DE, and vice versa. Hence, the number of features is duplicated because of cross-detection [1]. For the above time-domain features, F*_y_*_,1_ to F*_y_*_,18_ are extracted from the bearing signal of the local end collected by the sensor installed at the local end. F*_y_*_,19_ to F*_y_*_,36_ are extracted from the bearing fault signal of the local end collected by the sensor installed at the opposite end. The same below.

(2) Frequency domain: The original vibration signals are transformed into frequency signals using FFT. The frequency signals are divided into several bands, and the mean frequency (F*_y_*_,37_ and F*_y_*_,41_), root mean square of frequency (F*_y_*_,38_ and F*_y_*_,42_), frequency center (F*_y_*_,39_ and F*_y_*_,43_) and root variance frequency (F*_y_*_,40_ and F*_y_*_,44_) are calculated for each band. 

When the failure emerges, the energy distribution in the same bandwidth of different types of signals is different, and the energy distribution in different bandwidths of the same type of signal is also different. Therefore, the normalized energy of the selected IMF (F*_y_*_,45_ and F*_y_*_,46_) is extracted. Meanwhile, the singular value (F*_y_*_,47_ and F*_y_*_,48_) [14] is also extracted.

**Figure 6:**
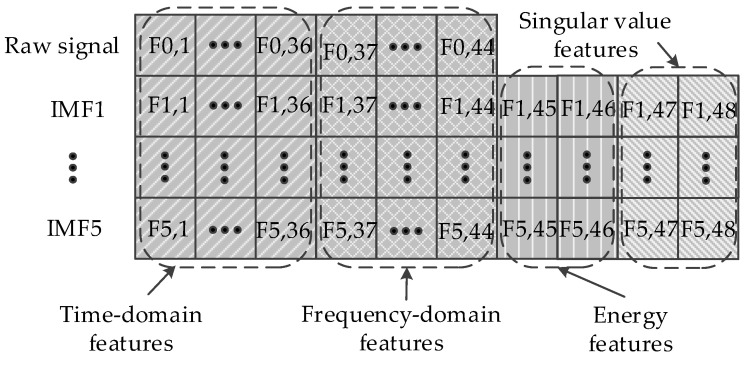
Distribution of features.

The time-domain and frequency-domain features are extracted from the raw signal and the selected IMF, and the normalized energy features and singular value features are extracted only from the selected IMF. The distribution of features is shown in Figure 6. Forty-four features, comprising 36 time-domain features and eight frequency-domain features, are extracted from the raw signal. The distribution of the time and frequency domain features is like the distribution in the raw signal; two normalized energy features and two singular value features are extracted from each IMF. Finally, 284 features are obtained from the feature extraction process.

## 6. Feature Analysis and Classification Ability Analysis of OCSVM and RF

The feature importance under different diagnosis targets and the classification ability of RF are analyzed in this section.

Three diagnosis targets are considered in this paper.

(1) Target 1: 4 types, including normal signals, ball fault, inner race fault, outer race fault;
(2) Target 2: 7 types, including normal signals and faults with different positions;
(3) Target 3: 19 types, including normal signals and faults with different positions and severities.

**Figure 7:**
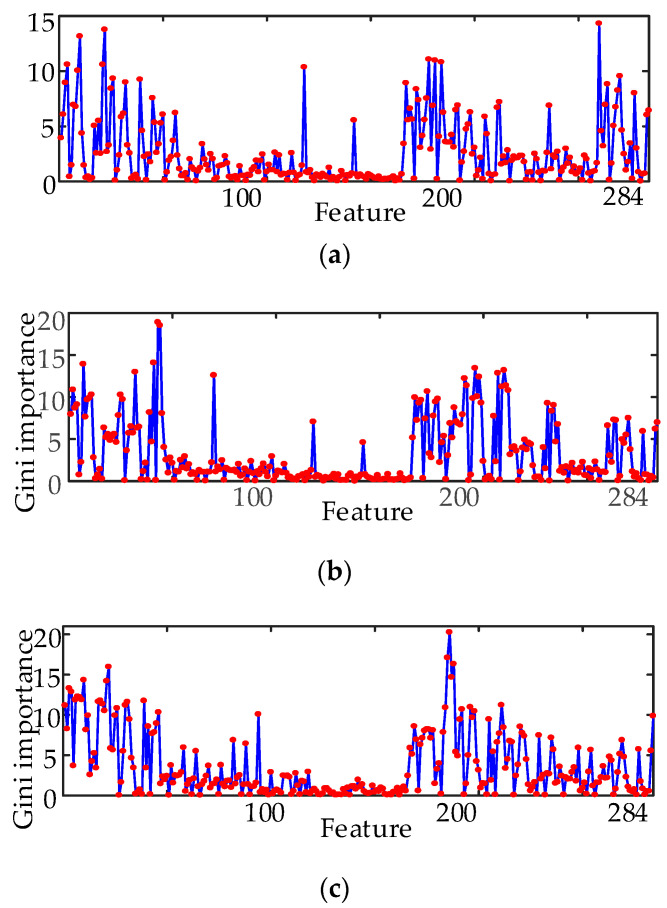
GI of all features under different diagnostic targets. (**a**) GI of all features under diagnostic target 1. (**b**) GI of all features under diagnostic target 2. (**c**) GI of all features under diagnostic target 3.

In the experiments, the entire dataset is divided into a training set, a validation set, and a test set. The training set comprises 60% of the entire dataset, and both the validation and test set comprise 20% of the entire dataset. Only the bearing faults diagnosed with different targets are classified by RF, the number of decision trees denoted by $n_{tree}$ is set at 500, and the feature number at each split denoted by $m_{\mathrm{try}}$ is set at 17. The GI of each feature for different diagnosis targets are shown in Figure 7. From Figure 7, we can observe that there is a great difference in the GI of various features for different targets. Feature No. 260 has the highest Gini importance, at 14.3, for target 1; Feature No. 46 has the highest Gini importance, at 18.9, for target 2; Feature No. 186 has the highest Gini importance, at 20.3, for target 3. 

**Figure 8:**
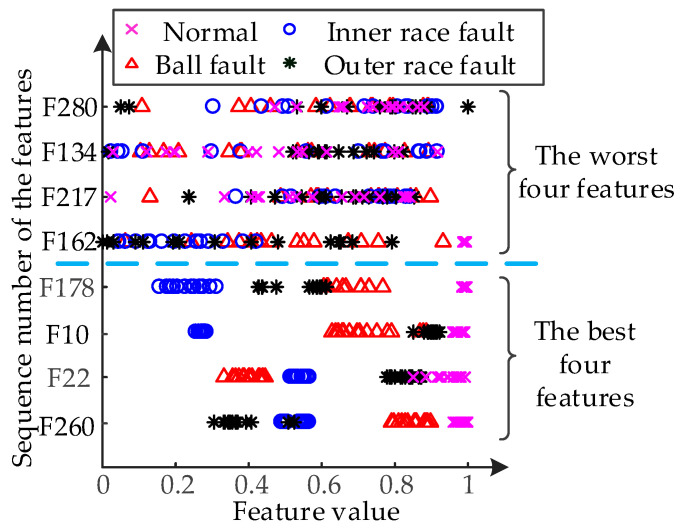
The feature value distribution of the first 4 features with the highest GI and the last 4 features with the lowest GI.

The feature value distribution of the first 4 features with the highest GI and the last 4 features with the lowest GI are also shown in Figure 8. From Figure 8, the feature value of the first four features for different types of signals only has a small scope of the cross-field, and their ability to distinguish the various faults is strong. The feature value distribution of the first four features has a large cross-field, and it is difficult to distinguish the various faults. This validates the effectiveness of the evaluation of the classification ability of the features using the GI. On the other hand, the importance of same feature for different diagnosis targets are different (as Figure 7). This means that the optimal feature set for different diagnosis targets will be different. 

**Figure 9:**
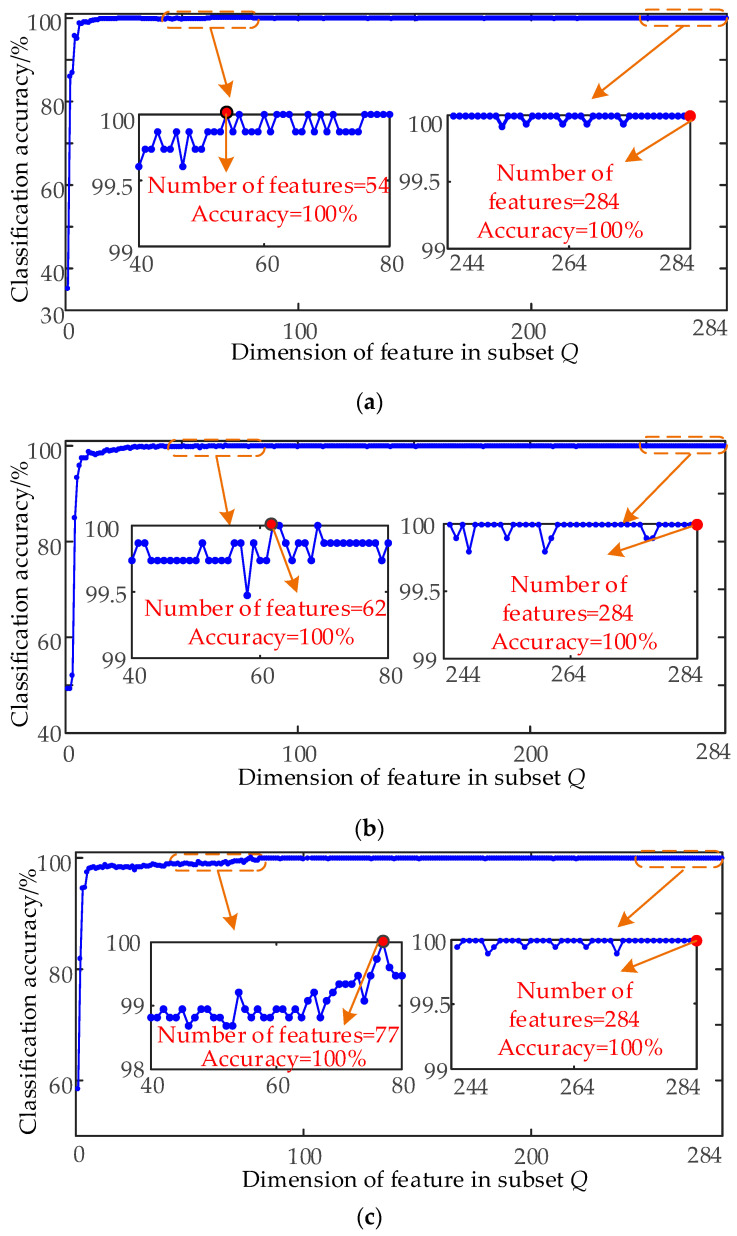
Classification accuracy of RF for various subsets. (**a**) Classification accuracy of RF for various subsets under target 1. (**b**) Classification accuracy of RF for various subsets under target 2. (**c**) Classification accuracy of RF for various subsets under target 3.

To improve the classification ability of RF, an experiment with different input feature sets is performed. The descending ordered 284 features by GI are added to an empty set *Q*. For each additional feature in *Q*, the new training set in *Q* is used to train an RF classifier, and the accuracy of the RF in the new test set is recorded. The classified accuracy of various subsets for diagnostic target 1, target 2 and target 3 are shown in Figure 9. 

Figure 9 shows that the classification accuracy of RF under various diagnosis targets gradually increases to 100% with the increase in feature number. After that, the classification accuracy of RF remains stable with the further increase of feature dimension. Therefore, RF can achieve high diagnosis accuracy with a high-dimensional original feature set for different diagnosis targets.

## 7. Diagnosis Result of Various Scenarios

The following three fault scenarios are set to verify the validity of the method proposed in this paper.

### 7.1. Fault Scenario 1: All Types are Included in the Training Set

To avoid the contingency caused by using only classification accuracy (*ACC*) as the measurement, a Kappa coefficient denoted by *K* is also used. The Kappa coefficient *K* is used to measure the consistency between the actual and predicted classifications. Considering both the Kappa coefficient *K* and classification accuracy can avoid the contingency when only considering the classification accuracy. The calculation method of *K* can be found in [31]. Therefore, the evaluation index denoted with η is as follows:

(12) $$\eta =\frac{ACC+K}{2}\times 100\%$$

To verify the classification ability of RF, a comparative test is carried out by OCSVM-RF, RF, SVM and BPNN, as shown in Table 1. The method of building the SVM and BPNN is the same as the method shown in [32]. Table 1 shows that RF has a better classification ability than BPNN and SVM for a high-dimensional original feature set. The diagnosis result of RF is decided by numerous decision trees and avoids false identification to the greatest extent. It is more suitable for high dimensional fault diagnosis scenario than other methods.

### 7.2. Fault Scenario 2: Samples of Various Fault Severities are Insufficient in the Training Set

In practical applications, samples with various fault severities are always insufficient and unbalanced. Traditional multi-classification methods may misidentify a sample with an unknown severity as the wrong type, even as a normal sample. Thus, the multi-classification method should first determine whether the mechanical state of the bearings is normal. 

To verify the fault diagnostic capacity of the proposed method when diagnosing a sample with unknown fault severity, the OCSVM-RF hybrid classifier is used for comparison with SVM, BPNN and RF. For OCSVM, v=0.75, σ=16.12. In this experiment, the fault location is regarded as the identified target. The ball fault of the bearing at DE denoted by DE-BAF is regarded as a special fault type with unknown fault severity; two types of fault severity samples with 50 samples per fault severity are randomly selected from DE-BAF as the test samples and not added in the training set. One hundred samples are randomly selected from the remaining kinds of fault severity in DE-BAF, which combines 100 normal samples and the remaining five fault types with 100 samples per type for constructing the training set. According to the feature set described in Figure 5, for the hybrid classifier, OCSVM is trained only by normal samples, and RF is trained using the remaining fault samples. SVM, BPNN and RF are trained by the entire training set. When the ball fault at FE denoted by FE-BAF is regarded as a special type, the training of the classifiers is the same as above. The classification results of the various classifiers for the special type are shown in Table 2.

As Table 2 shows, when the training set cannot completely cover the samples with various fault severities, SVM, BPNN and RF misidentify some samples with an unknown fault severity as the wrong type, even normal samples, illustrating that because of excessive reliance on the training samples, the state monitoring ability of multi-class classifiers is already weakened. The accuracy of RF is between 92% and 100%, and the accuracy of other classifiers is less than 87%. As compared with a single multi-class classifier, the accuracy of OCSVM-RF is 98% to 100%, and all test samples are identified as having a fault state. OCSVM-RF can retain the strong classification ability of RF while improving the ability of state monitoring.

## 8. The Comparisons of Diagnostic Results

Because the CWRU bearing dataset has been the benchmark in bearing fault diagnosis, the new method in this paper is used to compare with the methods proposed in the published papers, where all those methods are also using the CWRU dataset. The comparative results are shown in Table 3. In Ref. [2], a deep neural network for domain adaptation in fault diagnosis (DAFD) is proposed and applied to identify the four types of bearing faults, and finally a recognition accuracy of 94.73% was achieved. Amar et al. [5] used vibration spectrum imaging (VSI) and an artificial neural network (ANN) for bearings fault diagnosis and got 96.9% accuracy. In Ref. [4], a local connection network (LCN) constructed by normalized sparse autoencoder (NSAE), namely, NSAE-LCN, is used for bearing fault diagnosis, and 99.92% accuracy was obtained. Zhang et al. [9] used EEMD for feature extraction and an optimized SVM for the identification of six kinds of bearing faults, and they obtained 97.04% classification accuracy. In Ref. [19], EMD and wavelet kernel local fisher discriminant analysis (WKLFDA) are used for feature extraction and dimensional reduction, and SVM was used to classify ten bearing conditions. Finally, a classification accuracy of 98.80% was obtained. 

In all of the methods compared, only normal conditions and four to ten known fault types were selected to train multi-classifiers and carry out fault diagnosis. The influence of imbalance of samples is not considered in [2,3,4,5], [9] and [19]. Compared to other methods, the proposed method in this paper can detect the mechanical state of bearings correctly when the samples are imbalanced. Moreover, the training of classifiers is constructed according to the three different diagnostic targets, and the accuracy of the method is increased greatly. When the number of classes is eighteen, which is much more extensive and complicated than the number of classes usually found in related work, 100% classification accuracy is still achieved by the method proposed in this paper. Clearly, Table 3 shows that the new method has a superior ability to diagnose the bearing faults or an even more complicated mechanical system.

## 9. Conclusions

A bearing-fault diagnosis method that is based on a hybrid classifier and that considers the various diagnostic targets and imbalanced sample number is proposed.

The main contributions of this research are as follows:

(1) Common features in the field of bearing fault diagnosis are collected, and a comprehensive feature set is constructed.

(2) Various diagnostic targets based on a practical project were determined. RF with high dimensional comprehensive feature set are constructed, and optimal feature set and classifier structure are constructed in the training process with different diagnosis targets automatically. 

(3) The new method compensates for the shortcomings of misidentifying the fault type as normal samples for traditional methods under the scenarios with imbalanced training samples by a novel hybrid classifier constructed using OCSVM and RF combining the strong classification ability of RF and the state monitoring ability of OCSVM.

## Figures and Tables

**Table 1:** Classification results of different diagnosis targets.

Diagnostic Target	Classifier	*K*	*ACC*/%	*η/%*
target 1	OCSVM-RF	1	100	100
	RF	1	100	100
	SVM BPNN	0.9800 0.9750	97.50 97.50	97.750 97.500
target 2	OCSVM-RF	1	100	100
	RF	1	100	100
	SVM BPNN	0.9667 0.9683	97 97.25	96.835 97.04
target 3	OCSVM-RF	1	100	100
	RF	1	100	100
	SVM BPNN	0.9508 0.9250	95.75 95.25	95.415 93.875

**Table 2:** Classification results of various classifiers for samples with unknown fault severity.

Classifier	Test Fault Type	Test (Missing) Fault Level	Diagnosis Result		
			BAF	Other Fault Type	Normal State
SVM	DE-BAF	0.007, 0.014	79	17	4
		0.007, 0.021	86	8	6
		0.014, 0.021	82	18	0
	FE-BAF	0.007, 0.014	85	10	5
		0.007, 0.021	86	11	3
		0.014, 0.021	87	13	0
BPNN	DE-BAF	0.007, 0.014	79	10	11
		0.007, 0.021	76	13	11
		0.014, 0.021	80	20	0
	FE-BAF	0.007, 0.014	81	7	12
		0.007, 0.021	80	10	10
		0.014, 0.021	78	22	0
RF	DE-BAF	0.007, 0.014	93	3	4
		0.007, 0.021	98	0	2
		0.014, 0.021	100	0	0
	FE-BAF	0.007, 0.014	94	2	4
		0.007, 0.021	98	0	2
		0.014, 0.021	100	0	0
OCSVM-RF	DE-BAF	0.007, 0.014	97	3	0
		0.007, 0.021	100	0	0
		0.014, 0.021	100	0	0
	FE-BAF	0.007, 0.014	98	2	0
		0.007, 0.012	100	0	0
		0.014, 0.021	100	0	0

**Table 3:** Diagnostic results comparison of the literature and the new approach.

Ref.	No. of Classes	ACC/%	No. of Diagnostic Targets	Imbalance of Samples
[1]	19	98.13	1	not considering
[2]	4	94.73	1	not considering
[4]	4	96.90	1	not considering
[3]	10	99.92	1	not considering
[8]	6	97.04	1	not considering
[16]	10	98.80	1	not considering
Proposed method	4	100	3	considering
	7	100		
	19	100		

## Abbreviations

EMD	empirical mode decomposition
WPT	wavelet packet transform
LMD	local mean decomposition
EEMD	ensemble empirical mode decomposition
EWT	empirical wavelet transform
IMFs	intrinsic mode functions
CART	Classification and Regression Tree
RF	random forest
SVMs	support vector machines
BPNNs	back propagation neural networks
ELMs	extreme learning machines
DNNs	deep neural networks
DBNs	deep belief networks
CNNs	convolutional neural network
CWRU	Case Western Reserve University
VSI	vibration spectrum imaging
FFT	Fast Fourier transform
OCSVM	One-class support vector machine
EDM	electro-discharge machining
DE	drive end
DAFD	domain adaptation in fault diagnosis
ACC	accuracy
FE	fan end
AM-FM	Amplitude modulated-frequency modulated
ANN	artificial neural network
LCN	local connection network
WKLFDA	wavelet kernel local fisher discriminant analysis
